# An evaluation of the 'Designated Research Team' approach to building research capacity in primary care

**DOI:** 10.1186/1471-2296-9-37

**Published:** 2008-06-27

**Authors:** Jo Cooke, Susan Nancarrow, Jane Dyas, Martin Williams

**Affiliations:** 1 Trent Research and Development Support Unit, School for Health and Related Research, University of Sheffield, Sheffield, UK; 2 Centre for Health and Social Care Research, Sheffield Hallam University, Sheffield, UK; 3 Trent Research and Development Support Unit, Division of Primary Care, University of Nottingham, Nottingham, UK; 4 Trent Research and Development Support Unit, Department of Health Sciences, University of Leicester, Leicester, UK

## Abstract

**Background:**

This paper describes an evaluation of an initiative to increase the research capability of clinical groups in primary and community care settings in a region of the United Kingdom. The 'designated research team' (DRT) approach was evaluated using indicators derived from a framework of six principles for research capacity building (RCB) which include: building skills and confidence, relevance to practice, dissemination, linkages and collaborations, sustainability and infrastructure development.

**Methods:**

Information was collated on the context, activities, experiences, outputs and impacts of six clinical research teams supported by Trent Research Development Support Unit (RDSU) as DRTs. Process and outcome data from each of the teams was used to evaluate the extent to which the DRT approach was effective in building research capacity in each of the six principles (as evidenced by twenty possible indicators of research capacity development).

**Results:**

The DRT approach was found to be well aligned to the principles of RCB and generally effective in developing research capabilities. It proved particularly effective in developing linkages, collaborations and skills. Where research capacity was slow to develop, this was reflected in poor alignment between the principles of RCB and the characteristics of the team, their activities or environment. One team was unable to develop a research project and the funding was withdrawn at an early stage. For at least one individual in each of the remaining five teams, research activity was sustained beyond the funding period through research partnerships and funding successes. An enabling infrastructure, including being freed from clinical duties to undertake research, and support from senior management were found to be important determinants of successful DRT development. Research questions of DRTs were derived from practice issues and several projects generated outputs with potential to change daily practice, including the use of research evidence in practice and in planning service changes.

**Conclusion:**

The DRT approach was effective at RCB in teams situated in a supportive organisation and in particular, where team members could be freed from clinical duties and management backing was strong. The developmental stage of the team and the research experience of constituent members also appeared to influence success. The six principles of RCB were shown to be useful as a framework for both developing and evaluating RCB initiatives.

## Background

The current UK NHS R&D strategy, Best Research for Best Health [1] aims to establish the NHS as an internationally recognised centre of research excellence and this is being supported by significant reorganisation and investment in workforce development and research infrastructure. The strategy acknowledges that, in order to produce high quality research that informs policy and practice, it is important that many of those responsible for providing patient treatment and care are also research active. The need to develop the capacity of clinical practitioners to engage in health research has also been recognised in other countries, including Australia and the USA. [2-4].

Research capacity building (RCB) has been described as a "process of individual and institutional development which leads to higher levels of skills and greater ability to perform useful research" [5]. Unsurprisingly, the clinical areas most often cited as being of greatest need for increased research capacity are those with the lowest research skill and activity base, such as nursing, primary care, and the allied health professions [3,4,6]. Between 1995 and 2005, several policy initiatives were introduced in the UK to build research capacity in these groups, including the formation of Primary Care Research Networks (PCRNs) and Research and Development Support Units (RDSUs).

If it is accepted that an increase in the research capacity of under-researched clinical areas is desirable, then it is important to determine which methods are most effective for RCB in these settings. A number of papers have described different approaches to building research capacity, including training schemes and bursaries that supported practitioners to do research alongside practice [7-9]. Other initiatives invested effort in developing specific research skills in practitioners, for example writing for publication [10]. Investment in infrastructure to fund protected time for practitioners and research assistants has also been explored [11]. Fellowship training for primary care physicians has shown promising results in the traditional metrics of number of grants submitted, grants received, peer-reviewed publications and, more interestingly, in career progression [9,12].

Despite a growing body of literature about RCB, less is known about how to assess whether efforts to build research capacity have been successful. The research metrics most commonly used to assess RCB are journal publications and presentations at conferences [3,13,14], successful grant applications [3,7], and academic qualifications [8]. However, it has been argued that publications in peer-reviewed journals can be difficult for novice researchers to achieve [14] and, like grant successes, are only useful as mid- to long-term indicators of progress. Furthermore, traditional research metrics are only suitable for evaluating a subset of the many possible objectives of RCB initiatives. For example, the goals of RCB initiatives might include the ability to perform 'useful research' [5], to promote health gain for individuals and communities [3], or to support sustainable research activity [4]. In addition, there is recognition that research engagement can have a positive impact on job satisfaction [7,15], approach to everyday practice [3,7], and confidence building [8,10]. Consequently, it is argued that process measures are also important in assessing RCB [16] and that these may help to shed light on what happens inside the 'black box' of RCB interventions.

This paper describes the evaluation of a particular approach to research capacity building in primary and community care developed by Trent Research and Development Support Unit (RDSU) in the East Midlands and South Yorkshire area of England. The effectiveness of a 'Designated Research Team' (DRT) scheme to build research capacity in practitioner groups was evaluated using non-traditional process measures as well as traditional research outcome measures.

## Methods

The Trent RDSU DRT scheme was based on a programme originally developed by the Royal College of General Practitioners [11]. In the Trent RDSU version of the initiative, small teams of aspiring researchers from primary care, including general practitioners and other practice staff, allied health professionals, community nurses, social workers and pharmacists, were awarded £32,000 of funding over two years to enable protected time for up to three team members to engage in research. Additionally, DRT funding could be used to support skills development of team members, patient and public involvement in projects or project dissemination. Teams were structured in a variety of ways, but each included at least one member who was a novice researcher and one who was linked to an academic department. This requirement encouraged an apprenticeship system within the teams whereby more experienced researchers could support novices throughout the process. A range of RCB 'interventions' were utilised in the approach including:

### • Training

Formal research training was offered through short courses and structured research training programmes provided by the RDSU. Attendance of other external workshops and courses was funded from the DRT contract.

### • Mentorship

This was provided by regular meetings with the RDSU co-ordinator, and through the skill mix of the team. Mentorship is defined as a process whereby an experienced person (the mentor) guides another individual in the development and examination of their ideas, learning and personal and professional development [17].

### • Supervision

This was provided by the academic partner in the team, by periodic progress meetings with the RDSU, and as a component of some of the more structured educational programmes delivered by the RDSU. The function of supervision was to monitor the progress of research projects.

### • Partnership development

The teams were encouraged to be outward looking and to build research partnerships outside the team.

### • Protected time from clinical work

Protected time for research was supported through DRT contracts that paid other clinicians to undertake clinical sessions of team members.

Teams were accountable at six-monthly meetings, at which they reported to the RDSU on their progress against pre-agreed, team specific, objectives. These objectives were developed and integrated into DRT contracts that specified, for example, timeframes for the development of a research idea into a protocol, for achieving ethical approval, for the collection or analysis of project data, for skills development of team members or for dissemination activities (such as conference presentations or research publications). Objectives were negotiated separately with each team, based on their developmental stage, to ensure that they were both challenging and achievable. Table 1 provides a brief description of the six teams that were given Trent RDSU DRT awards and associated support between 2000 and 2004.

**Table 1:** Description and history of DRTs.

**Team**	**Initial members of DRTs**	**Academic support**	**Team background**	**Team membership during DRT period**
**1**	Two senior academic GPs, a medical statistician, nursing and GP practice staff from 2 GP practices. A clinical governance lead from a Primary Care Trust (PCT).	Academic general practice at a local university.	Experienced members of the team were researchers who worked well together and used DRT funds to maintain this.	The team had the same constituents throughout the funding.
**2**	Five GPs from four practices, one district nurse, one operations manager in primary care from social services.	Nursing at a local university.	The team evolved out of a steering group of motivated practitioners looking at health inequalities based in a Health Action Zone (HAZ).	The team members expanded over time, and related to the projects undertaken, working in an interdisciplinary way.
**3**	GPs and practice staff across two GP surgeries including a range of practitioners (community nurses, health visitors and a nurse practitioner) and a general practice manager.	Academic general practice.	The practitioners had little experience of doing research, but one had gained funds from an NHS research programme for a project developed with the RDSU.	The core parts of the DRT remained the same although another GP joined the group as the nurse practitioner transferred to his surgery, and an attached health visitor transferred to another practice.
**4**	A uni-professional team of podiatrists, including the head of service, a practice facilitator and a foot care assistant.	Two universities from academic podiatry.	The team were based in an NHS department of podiatry, which was research active.	The team had the same constituents throughout the funding.
**5**	GP, a clinical psychologist, and a PCT executive manager.	Health service researchers in school of Health and Social Care.	Evolved from a strategy group in the PCT. The PCT aimed to become a Teaching PCT, and to include the building of a research culture as part of their application.	The team members expanded over time, and related to the projects undertaken.
**6**	Comprised of community pharmacists, a pharmacy project facilitator and a GP.	Academic general practice at a local university.	Evolved from a group of practitioners exploring issues of prescribing in primary care.	The team had the same constituents throughout the funding, although some members did not attend meetings. This team did not complete a research project and DRT funding was withdrawn.

The evaluation of the DRT initiative drew on a framework of constructs developed previously by Cooke [18]. This framework is presented below in the form of six propositions against which the DRT scheme was evaluated:

Proposition 1: The DRT approach was effective at building skills and confidence

Proposition 2: The DRT approach helped to ensure that research was 'close to practice'

Proposition 3: The DRT approach facilitated the development of linkages and partnerships

Proposition 4: The DRT approach supported appropriate research dissemination

Proposition 5: The DRT approach helped to build sustainability and continuity

Proposition 6: The DRT approach lead to improvements in research infrastructure investment

For each proposition, a set of process and outcome indicators were derived (Table 2). These were identified from the literature on RCB across a range of interventions, from the experience of members of the RDSU working with practitioners and through a structured workshop facilitated by the RDSU with research support workers and managers in healthcare.

**Table 2:** Principles for research capacity building and associated indicators.

**Principles**	**Indicators**
1. Research capacity is built by developing appropriate skills, and confidence, through training and developing the opportunity to apply skills in practice	• Skills developed through completing project
	• Evidence of career development
	• Evidence of confidence building (sharing skills, applying skills to new situations, working with other professional groups in research)
	• Completed training
	• Completed/working towards research qualifications
2. Research capacity building should support research 'close to practice' to highlight its usefulness to practice	• Research question developed from practice
	• Research question developed with patients and the public
	• Examples of research projects, and findings, have had an impact on local practice
3. Developing linkages, partnerships and collaborations enhances research capacity building	• Evidence of links between practice and universities
	• Evidence of links between practitioners in research
	• Evidence of inter-professional working
4. Research capacity building should ensure appropriate dissemination	• Local dissemination
	• Conference publications
	• Peer-reviewed publications
5. Research capacity building should ensure elements of continuity and sustainability	• Applications for funding
	• Successful grant applications
	• At least one DRT member continued to work in research after DRT finished
6. Developing appropriate infrastructure enhances research capacity building	• Support infrastructure established in team administration
	• Links developed to management in organisation
	• Arrangements were in place for team members to take protected time to undertake research

A data collection template (Table 3), referencing these domains and indicators, was developed to support assessment of whether or not (or the extent to which) teams had shown evidence of RCB under the DRT scheme. The template was used by the four researchers (all Trent RDSU co-ordinators) to collect and collate information about each team. Multiple sources of data were used to complete the template, including minutes of meetings, written reports (provided every six months by the DRT to the RDSU), notes from discussions with RDSU co-ordinators, reflective sessions with the teams, and feedback from DRT leads. Where investigator views differed, discussions took place and supporting evidence examined until consensus was reached. A case report was prepared for each DRT which was then sent to the DRT lead to check for completeness and accuracy.

**Table 3:** Data collection pro-forma.

**Brief history of the team's development**
**Organisational structure/structures they include**

**Team description**
What is the professional mix/research skill mix of the team?
Who is the academic support?
- Which dept/university did they come from?
- What do they bring to the team?
- How much commitment did they show?

**Protected time and arrangements for clinical duties to be covered by another person**
Did the money buy protected time?
How was clinical cover arranged?

**Training**
What type of training was utilised?
Have you got any sense of what was gained through the training?
How were the training needs identified?
How timely was the training given?
Did all members of the team utilise the training budget?
Who used it and how?
Was any outreach training done, if so, by whom and on what topic?

**Mentoring and supervision**
Who provided mentoring and supervision?
What was the role of the RDSU co-ordinator in this?

**Project**
Did the team have a joint project/several projects on application?
Describe projects undertaken by the team.
What did each team member contribute to the project?
What evidence is there of project management?

**Process indicators**
How often did the team meet?
Who attended the meetings?
How were jobs delegated and how was this communicated between team members?
What level of commitment to complete allocated tasks was shown?
Who took the lead?
What is the nature of this leadership?
What personal qualities are evident in the leadership styles?

**Close to practice**
How relevant was this to primary care local and national context?
Were patients and the public involved?

**Outcomes/dissemination**.
What outcomes were agreed with the DRT?
Were they met? (Include – submissions, successful publications, conference presentations, fellowships, career developments)
Any innovations in dissemination?

**Research culture**
Was research valued/expected/enjoyed in the context of the working context of the team?
Was EBP evident?

**Linkages**
Were any collaborations developed outside the DRT?
If so, how did these come about?
Networks – what sort of networking did the team undertake?
What were the spin offs from this networking?

**Sustainability and continuity**
What happened after DRT funding stopped?
How did the RDSU keep contact/keep motivation up?
How well are the team members engaged with other networks?
Does the DRT still function as a team?

**Were there any surprises?**
**What was the input of the RDSU?**

## Results

Findings on whether capacity development was indicated by the teams within each of the six domains are summarised below.

### Proposition 1: The DRT approach is effective at building skills and confidence

All of the teams met at least one of the indicators of building skills and confidence (see Table 4). The most frequently achieved indicator was that of gaining skills through training, either provided by the RDSU, or from other sources. Many of the novice team members attended a ten-day introductory course run by the RDSU, during which participants undertook a small research project. More experienced researchers accessed bespoke research training paid for with DRT money. Some team members undertook and completed research qualifications, including MSc, MD and PhD qualifications.

**Table 4:** Performance against indicators of skills and confidence.

**Indicator**	**Teams**						**Comments**
	**1**	**2**	**3**	**4**	**5**	**6**	

Skills developed through completing project	✓	✓	✓	✓	✓		Team 6 were unable to decide on a project and funding was eventually withdrawn.
Evidence of career development	✓	✓	✓	✓	✓		
Evidence of confidence building (sharing skills, applying skills to new situations, working with other professional groups in research)	✓	✓	✓	*	✓		* Team 4 were uni-professional. They worked internationally with other podiatrists, but did not work with other disciplines in research.
Completed training	✓	✓	✓	✓	✓	✓	All teams had at least one member who attended RDSU training. Teams also had 'on-site' training by RDSU co-ordinators.
Completed/working towards research qualifications		✓	✓	✓	✓		

Five of the teams were able to conduct and complete a project during the lifetime of the DRT, and members were able to develop research skills by the experience of doing research. The successful completion of projects was linked to a clearly articulated project idea aimed at improving practice, problem solving, or promoting professional development. Organisational support appeared critical in successful completion of research.

Team four used a multi-method project design on workforce flexibility that included documentary analysis, questionnaires, interviews and focus groups with stakeholders [15]. Each team member took the lead on a specific component of the project and developed their understanding of one methodology in depth. The team felt that the project was successful because a range of different, but complementary research skills were planned and developed in the team.

Teams two and five used the DRT funding to co-ordinate and expand existing programmes of work. Both teams developed from Primary Care Trust (PCT) research or practice planning groups. Team five developed from a research strategy group, and had strong links with the PCT executive and clinical audit group. Many projects were linked to the strategic aims of the PCT, and to clinical issues arising from clinical audit. Both approaches co-opted extra practitioners into the 'team' relating to different project needs, with a DRT member becoming the research lead on projects conducted. In this way the team grew and drew in others in the Trust as the number of projects expanded. This approach also meant that the original DRT members developed research management experience, and developed the skills of others in their organisation to build capacity.

Team six were unable to start a project. They were enthusiastic and wanted to work together on research, but had not formulated a focussed research idea prior to receiving the DRT funding. As a result, initial targets were not met and funding was withdrawn at an early stage. Team three also had difficulty focusing on a joint team project, but were able to use the DRT funding to complete other projects, and to undertake one small project developed by two team members. Both Teams three and six spent time searching the literature to formulate their idea. Critical appraisal skills, and therefore research capacity, was developed in these teams, but this left little time for the research project itself. This was compounded in both teams because of a lack of locum cover to allow team members to be freed from clinical duties.

Learning and skill development also occurred through an apprenticeship approach, facilitated by the range of experience in team members. Ongoing mentorship was also provided by the RDSU co-ordinators. The level of input from academic DRT members varied, with a high level of commitment having a positive impact on research productivity.

### Proposition 2: The DRT approach helps to ensure that the research is 'close to practice'

The five teams that were able to complete projects, performed well against two of the indicators, reflecting closeness to practice (Table 5). All research projects conducted by the DRTs demonstrated relevance to practice. Three types of projects were undertaken:

**Table 5:** Performance against 'close to practice' indicators.

**Indicator**	**Teams**						**Comments**
	**1**	**2**	**3**	**4**	**5**	**6**	

Research question developed from practice	✓	✓	✓	✓	✓		Five teams were able to do project work that related to practice. Team six were unable to develop an idea together.
Research question developed with patients and the public		✓		*	*		* Teams four and five worked with patients and the public but not on research questions. Team two developed research priorities with patients and the public.
Examples of research projects, and findings, have had an impact on local practice		✓	✓	✓	✓	✓	Team five linked projects in with clinical audit.

#### • Clinical practice research

e.g. effectiveness of cardiac rehabilitation; the management of depression in primary care.

### • Service delivery research

e.g. workforce flexibility and extended clinical roles; developing models for intermediate care.

### • Continual professional development research

e.g. exploring GP trainers' barriers to teaching evidence based practice to medical students; evaluating training packages for GPs.

Team five were particularly successful in this regard and linked their research to quality improvement cycles within the PCT. Here research priorities identified from clinical audit developed into research projects. The results of the research were fed back to practice within the organisation, for example the research impacted on practice protocols around increasing influenza and pneumococcal vaccination rates in high-risk groups. This team included senior management in the PCT, and was part of a Teaching PCT with organisational objectives linking research to a learning environment and quality improvement [19].

There was evidence of research influencing practice in most of the teams where a research project was undertaken. This particularly occurred where the research project highlighted gaps in information, or inadequacies in practice, and where the team planned a course of action to remedy this. Examples include developing patient information about total knee replacement because research interviews had highlighted inconsistencies in information-giving (Team three). Team two developed a self-help group for patients from Afro-Caribbean communities as a consequence of information gathered in research projects that highlighted a need to support this patient group. Practitioners also talked about the 'spin offs' on their every day practice. This was usually in the form of applying evidence to their practice as a consequence of reading literature relating to their study, or of information they acquired at conferences.

User involvement was poorly developed in most teams and only Team two developed significant research capacity in this area. They conducted focus groups with people from ethnic minority communities to identify research priorities and develop research projects [20]. This team were highly motivated and concerned about health inequalities in the geographical area they worked in, and were better networked to support community involvement than many of the other teams.

### Proposition 3: The DRT approach facilitates the development of linkages and collaborations

The indicators for this principle were achieved by all the teams (see Table 6). This was not surprising, as linkages and collaborations were inherent in the DRT model, and included those between researchers and universities, clinicians and researchers, and between practitioner groups. Expert methodological advice was available to the teams from the RDSU, who also facilitated links to local research networks. Team three were able to extend their networks internationally and attracted funding to compare workforce issues in Australia and North America with those in the UK. The 'organic' approach adopted by Teams two and five actively involved practitioners from outside the original DRT on projects, which seems to have had an impact on the research culture of the wider organisation. This is described further elsewhere [19]. Linkages and collaborations were important components of longer term sustainability as they provide a basis for continuing research and attracting further funding.

**Table 6:** Performance against linkages and collaboration indicators.

**Indicator**	**Teams**						**Comments**
	**1**	**2**	**3**	**4**	**5**	**6**	

Evidence of links between practice and universities	✓	✓	✓	✓	✓	✓	All teams had links with universities through the RDSU and academic members of the team.
Evidence of links between practitioners in research	✓	✓	✓	✓	✓	✓	The DRT model worked with practitioners doing research alongside practice.
Evidence of inter-professional working	✓	✓	✓		✓	✓	Team four remained uni-professional in their research project.

### Proposition 4: The DRT approach supports the development of appropriate research dissemination

All of the teams who received funding for the full two-year term were able to demonstrate achievement against the dissemination indicators (see Table 7). This was facilitated by each team having specific DRT objectives around this indicator, which included local dissemination, and more traditional outcomes of dissemination in peer-reviewed publications and conference presentations. Teams varied in the number of traditional outcomes they were able to achieve (Table 8). Four of the six teams had publications related to projects conducted during the funding period, and another used the funding to write up other projects (Team three). Teams with high numbers of publications did not always reflect progress for novice researchers. Those teams which had a planned dissemination strategy, which included novice researchers, showed greater impact in this regard.

**Table 7:** Performance against dissemination indicators.

**Indicator**	**Teams**						**Comments**
	**1**	**2**	**3**	**4**	**5**	**6**	

Local dissemination	✓	✓	✓	✓	✓		
Conference presentations	✓	✓	✓	✓	✓		An objective set for each team was around conference presentations. This included specific objectives for novice researchers.
Peer-reviewed publications	✓	✓	✓ *	✓	✓		* Some members of Team three completed publications on project work that was undertaken before DRT funding. Funding allowed protected time to do writing.

**Table 8:** Dissemination outputs at time of the evaluation.

	**Peer-reviewed publications accepted/published**	**Conference presentations**
Team 1	15	8
Team 2	4	11
Team 3	2	3
Team 4	3	10
Team 5	16	5
Team 6	-	-

Other dissemination successes included the use of research to inform strategy, both locally and regionally. The leader of Team four was seconded to the Workforce Development Confederation, which is a regional organisation whose function is related to workforce planning. The insights developed in the DRT research project helped to inform some of the work undertaken during this secondment. Two other projects used research information to develop practitioner training packages, one being disseminated in CD-Rom format.

All teams disseminated their work locally.

### Proposition 5: The DRT approach helps to build sustainability and continuity

At the time of publication at least one member of each team who received the full award, continued to work in research (see Table 9), through maintaining links with the RDSU, or by being part of research networks, academic departments, or through further funding. Four of the six teams achieved external funding. The total amount gained by the teams was £259,750. This ranged from £5,000 for Team five to £144,450 for Team one.

**Table 9:** Performance against continuity and sustainability indicators

**Indicator**	**Teams**						**Comments**
	**1**	**2**	**3**	**4**	**5**	**6**	

Applications for funding	✓	✓	✓	✓	✓		Applying for external funding was an objective set for all teams.
Successful grant applications	✓	✓		✓	✓		The size of the grant captured varied from £5000 for Team five to £144, 450 for Team one.
At least one DRT member continued to work in research after DRT finished	✓	✓	✓	✓	✓		The relationships built through the DRT continued, and team members worked as collaborators, particularly with the RDSU on projects and in research networks.

### Proposition 6: The DRT approach leads to improvements in research infrastructure investment

See Table 10 for achievement against the infrastructure indicators. Having protected time to do research appeared to be a key determinant to the successful completion of research. Because of difficulties in being freed from clinical duties, funding could not always be used to provide protected research time. This was found to be a particular problem for independent practitioners such as community pharmacists and GPs, where locum cover was difficult to arrange. This was also a concern for some nursing staff in Team three. However, four of the six teams were able to use funds to provide locum cover and find protected research time from clinical work. One team addressed poor locum cover arrangements by extending part time working hours funded by the DRT monies.

**Table 10:** Performance against infrastructure indicators.

**Indicator**	**Teams**						**Comments**
	**1**	**2**	**3**	**4**	**5**	**6**	

Support infrastructure established in team administration			✓	✓	✓		Administrative support helped with ethics and governance applications. Minutes taken in meetings by administrators helped action planning.
Links developed to management in organisation		✓		✓	✓		Teams that included managers, enabled protected time for practitioners. In Team five, management links enabled connections to quality improvement cycles in the PCT.
Arrangements were in place for team members to take protected time to undertake research	✓	✓	*✓	✓	✓		Having someone to cover clinical duties whilst engaged in research was a key determinant of enabling protected time for research to be undertaken. * Nurses had difficulty in Team three, and finding locum cover was a particular problem for the pharmacists in Team six. A GP in Team three had difficulty obtaining locum cover but was able to achieve protected time to do research by extending her part time hours.

Tensions between spending time on research and other professional development needs were sometimes apparent. For example, a nurse in Team three undertook a professional qualification related to nurse prescribing. This was considered more relevant by their manager than doing research or research training and so was more strongly encouraged.

## Discussion

This paper describes the DRT approach to RCB in six teams supported by Trent RDSU through funding, mentorship and expert support, and it's evaluation against an RCB framework developed by Cooke [18]. The evaluation shows that, in general, the DRT approach was effective in developing the research capabilities of supported teams. It proved particularly effective in developing linkages, collaborations and skills. The evaluation drew on both quantitative data (such as the number of projects completed, papers written, grant applications, training courses undertaken, etc) as well as qualitative judgements about whether RCB had taken place (such as evidence of linkages with practitioners and academics or with the management in their organisations). Although some of the data was based on the retrospective views of the RDSU co-ordinators who worked with the teams, with the associated risks of selection and recall bias, the majority of findings were supported by multiple data sources, including contemporary documentation. Findings were fed back to the lead applicants of the DRTs for confirmation and verification.

While most teams demonstrated promising research capacity development, we should be cautious in attributing this progress solely to the DRT scheme. The RDSU purposefully selected teams who were enthusiastic about doing research, and progress may be related to their enthusiasm and tenacity. We had no control groups of unsupported but enthusiastic groups with which to compare. Variations in success around different indicators may also highlight intrinsic differences within the teams, for example Team two's success in working with ethnic minority communities and enabling user involvement is likely to be rooted in their motivation around inclusion and health inequalities, rather than the DRT model. Nevertheless, the DRT approach appears to provide opportunities for enthusiasts to develop, and this was a common theme expressed by team members.

The DRT is complex in that it includes a range of RCB 'interventions' such as training, mentorship, supervision, partnership development and protected time from clinical work. It is however, flexible in that support packages and expectations can be tailored to individual teams.

This evaluation suggests that the DRT approach is effective in promoting change and development across most RCB domains, and is particularly strong in developing linkages, collaborations and skills. It also highlights the potential synergy and interactions between principles that may impact on success. It is possible, for example, that development of linkages and collaborations could impact on sustainability. Many of the partnerships and linkages developed during the DRT funding period continued after the funding stopped. Sustainability is an important outcome for RCB, particularly in the context of current UK policy which aims for individual career progression. This finding has implications for future RCB interventions which should consider incorporating collaborative support to strengthen long term impact. Others have recognised that building trusting relationships to enhance 'social capital' [21], is critical for capacity building. This concept and its' associations with RCB effectiveness warrants further investigation.

The DRT approach promotes research skills development through education, training, mentorship and opportunities to apply new research skills. Teams with links to strategy and organisational planning groups engaged practitioners outside the team, and so developed skills in the wider organisation. When developing the DRT model further, it may be useful to measure not only the skills development in the teams, but also those in their wider organisations.

The DRT model encourages and supports researchers to complete the full research cycle, including appropriate dissemination. Five of the teams published in peer-reviewed journals, with three teams producing a first publication by novice researchers. These outputs related to setting specific objectives for the teams with strong academic mentorship. Although these are demanding outputs for novice researchers, it seems that, with appropriate mentorship and support, such outcomes are attainable. Publication is likely to have an impact on the sustainability of the research careers of team members by building a publication track record.

The DRT approach showed mixed success in the principle relating to 'producing research that is close to practice', although several examples of developing practice-relevant research were evident. Other benefits included the use of research evidence in practice, and other 'spin off' service developmental activity (e.g. better patient information). The impact of RCB on the quality of the service seems an important area for further investigation.

There was only very limited evidence that the DRTs had supported the development of closer links with patients and the public in research. The reason for this is unclear but it may be that new researchers are less confident to include patients and the public in the research process whilst they themselves are still learning.

The DRT approach was not uniformly successful for all teams who were initially funded. Two factors seemed to be particularly important in RCB when comparing the more productive teams (one, two, four and five) with those who showed least progress (three and six).

Firstly, the DRT approach appeared to require that some team members had previous experience of undertaking research and/or research expertise. Novice team members appeared to develop as researchers more quickly when working with more experienced peers. At the outset, groups were at very different developmental stages as 'research teams' and this was reflected in their subsequent progress. For example, Team six, who were unsuccessful in developing a research idea, had little research experience and had not previously conducted any research together as a team. They became stuck at the research planning stage and were unable to progress. Others have highlighted the developmental nature of primary care research teams [22] and the findings from this evaluation support this observation. This finding suggests that greater value may be gained in supporting teams who already have a track record of research collaboration, even if this is fairly minimal.

Secondly, the culture of the host organisations in which the teams were located appeared to influence the team's productivity and research impact. It was clear that poor organisational support was often associated with a lack of protected research time (this was also true of independent practitioners who had similar difficulties in setting time aside to do research). Teams that were embedded in organisations where research was valued, or where the research activity linked to quality cycles within the organisation, produced more outputs and these were more likely to be implemented. It is also pertinent that the more successful DRTs included managers who were able to establish protected research time for team members.

Congruence between the objectives of the team and those of the organisation also seemed to influence success. For example, Team five were situated in a PCT that was seeking 'teaching PCT' status. Having research-active practitioners contributed to the teaching PCT application. Similarly, Team four helped to realise the vision of the podiatry departmental manager to make the department research active. Others have highlighted the importance of matching RCB initiatives to those of the organisation [23], and this also resonates with recent UK policy which highlights the need for developing a research culture within NHS organisations [1], which links research to quality improvement and professional development [24]. These findings suggest that RCB initiatives should also consider the organisational context of individuals or teams they intend to support.

Using the principles as an evaluative framework for RCB, has demonstrated the value of using a blend of less traditional process measures and more traditional 'hard' outcomes. Some of these non-traditional measures have captured local impact on practice, organisational development, and have highlighted the importance of linkages and collaborations. These principles could be further developed as a tool for measuring research capacity which may be useful to examine the effectiveness of other RCB activities, such as grants, fellowships, and research bursaries, which could form a basis for comparison of the effectiveness of different capacity building approaches.

This research has also shown that reliance on the more traditional outcome indicators alone are not necessarily an accurate reflection of the effectiveness of particular approaches to capacity building, and that a fuller picture is developed using the RCB principles framework.

## Conclusion

The DRT model was shown to be effective in building research capacity. It builds skills for clinicians to engage in research, through education, training and applying research skills in practice, and provides opportunities to sustain research activity through developing supportive linkages and collaborations. It has also been successful in producing traditional outcomes of peer-reviewed publications, and conference presentations, but has also had some influence on practice and organisational change. DRT members have suggested that doing research has affected how they work in practice, and on their use, and access to research evidence. The context and culture of the organisation in which the team is placed, and developmental readiness of the team seem to be important factors in influencing progress and impact. Additionally, the RCB framework described in this paper, has the potential to form a valuable basis for evaluating and comparing a range of research capacity building approaches.

## Abbreviations

CD: Compact Disc; DRT: Designated Research Team; EBP: Evidence Based Practice; HAZ: Health Action Zone; GP: General Practitioner; PCT: Primary Care Trust; RCB: Research Capacity Building; RDSU: Research Development Support Units; UK: United Kingdom; UK CRN: United Kingdom Clinical Research Network.

## Competing interests

The authors declare that they have no competing interests.

## Authors' contributions

JC co-ordinated the paper, SN, JD and MW gave final approval of the submitted draft, contributed to the drafting of the manuscript, revising, and theoretical development of the manuscript.

## Pre-publication history

The pre-publication history for this paper can be accessed here:



## References

[B1] Department of Health (2006). Best Research for Best Health: A New National Health Research Strategy.

[B2] Department of Health (2004). Research Capacity Development Strategy.

[B3] North American Primary Care Research Group (2002). What does it mean to build research capacity?. Family Medicine.

[B4] Albert E, Mickan S (2002). Closing the gap and widening the scope. New directions for research capacity building in primary health care. Australian Family Physician.

[B5] Trostle J (1992). Research Capacity building and international health: Definitions, evaluations and strategies for success.. Social Science and Medicine.

[B6] Mant D (1997). National working party on R&D in primary care. Final Report..

[B7] Lee M, Saunders K (2004). Oak trees from acorns? An evaluation of local bursaries in primary care.. Primary Health Care Research and Development.

[B8] Bateman H, Walter F, Elliott J (2004). What happens next? Evaluation of a scheme to support primary care practitioners with a fledgling interest in research.. Family Practice.

[B9] Ried K, Farmer EA, Weston KM (2007). Bursaries, writing grants and fellowships: a strategy to develop research capacity in primary health care.. BMC Family Practice.

[B10] Ried K, Fuller J (2005). Building a culture of research dissemination in primary health care: the South Australian experience of supporting novice researchers. Australian Health Review.

[B11] Smith LFP (1997). Research general practices: what, who and why?. British Journal of General Practice.

[B12] Taylor JS, Friedman RH, Speckman JL, Ash AS, Moskowitz MA, Carr PL (2001). Fellowship training and career outcomes for primary care physician-faculty. Academic Medicine.

[B13] Del Mar C, Askew D (2004). Building family/general practice research capacity. Annals of Family Medicine.

[B14] Campbell SM, Roland M, Bentley E, Dowell J, Hassall K, Pooley J, Price H (1999). Research capacity in UK primary care. British Journal of General Practice.

[B15] Cooke J, Nancarrow S, Hammersley V, Farndon L, Vernon W (2006). The "Designated Research Team" approach to building research capacity in primary care. Primary Care Research and Development.

[B16] Carter YH, Shaw S, Sibbald B (2000). Primary care research networks: an evolving model meriting national evaluation.. British Journal of General Practice.

[B17] Standing Committee on Post-graduate Medical and Dental Education (1998). An enquiry into mentoring supporting doctors and dentists at work.

[B18] Cooke J (2005). A framework to evaluate research capacity building in health care. BMC Family Practice.

[B19] Dyas J, Moody L, Siriwardena N (2005). Improving research quality and health care in rural areas: a case study of the contribution of a teaching primary care trust. Quality in Primary care.

[B20] Brown K, Dyas J, Chahal P, Khalil Y, Riaz P, Cummings-Jones J (2006). Discovering the research priorities of people with diabetes in a multicultural community: a focus group study. British Journal of General Practice.

[B21] Griffiths F, Wild A, Harvey J, Fenton E (2000). The productivity of primary care research networks. British Journal of General Practice.

[B22] Macfarlane F, Shaw S, Greenhalgh T, Carter YH (2005). General practices as emergent research organizations: A qualitative study into organizational development. Family Practice.

[B23] Joffres C, Heath S, Farquharson J, Barkhouse K, Latter C, MacLean DR (2004). Facilitators and challenges to organizational capacity building in heart health promotion. Qualitative Health Research.

[B24] Lansang MA, Dennis R (2004). Building capacity in health research in the developing world. Bulletin of the World Health Organization.

